# The Ergonomic Knowledge and Practice of Dental Students in a Tertiary Institution in South Africa

**DOI:** 10.1155/2022/4415709

**Published:** 2022-07-20

**Authors:** U. K. Moosa, A. Bhayat

**Affiliations:** School of Dentistry, University of Pretoria, Pretoria, South Africa

## Abstract

Proper ergonomic practices are crucial practices to be considered when working on dental patients, and are often neglected during the dental students' training. We aimed to assess the dental students' knowledge of ergonomics as well the prevalence of their musculoskeletal pain. *Methods*. A cross sectional analytical study was conducted at a dental school in South Africa. The sample included all senior dental, and dental hygiene students registered in the 2021 academic year. A modified questionnaire using an online platform assessed the student's level of practical and theoretical knowledge of ergonomics; their personal assessment of their competency in implementing ergonomics; and their prevalence of musculoskeletal pain. The knowledge scores were calculated to determine the overall scores. Data was analysed using the Statistical Package for the Social Sciences (SPSS). Ethical clearance was obtained from the Faculty Ethical Committee and all information was anonymous. *Results*. The response rate was 52% (*n* = 106), the mean knowledge score was 68%, and 53% reported to be able to successfully implement their ergonomic knowledge practically. The prevalence of musculoskeletal pain increased from 32% prior to entering dental school to 78% during dental school. Pain was most commonly reported to be on the back (77%), neck (51%), and shoulders (51%). *Conclusion*. The majority of students had an average level of knowledge regarding ergonomic principles, however, the practical application was poor. Many students reported to have suffered from back pain which seemed to have started since treating patients. The supervisors should educate and assist students to practice healthy ergonomic postures during clinical and pre-clinical sessions.

## 1. Introduction

Studies have shown, that despite the importance of the implementation of correct seating posture, many practitioners fail to implement it in practice [1]. This has been ascribed to a number of reasons which include: inadequate emphasis during training at dental school and a general disregard of one's posture on account of the sheer difficulty in reaching the tiny spaces of the mouth [1]. Negligent posture is one of the reasons for the prevalence of musculoskeletal disorders amongst dentists [1]. Another factor that is becoming increasingly prevalent in causing musculoskeletal disorders, is the increasing dependence on electronic devices such as cell-phones and laptops [2]. Such impediments have had a vast negative impact both on the quality of life of the practitioner, as well as on the quality of the health-care delivered by practitioners [3]. Fatigue and muscle strain in the neck, shoulders and back, as well as the hands and wrists due to muscle strain, are some of the physical consequences [3]. Furthermore, these effects have an impact on the workplace efficacy and productivity of the practitioner [3]. Ideally, a dentists' working posture should be relaxed, balanced and comfortable, without severe strain of any sort [3]. However, studies have reported that not enough attention has been given to the implementation of this neutral working position during the training of dental students [4, 5]. As a result, the authors of these studies have recommended, that the correct seating postures and neutral working position should be regularly taught and assessed during the undergraduate training program. One such study reported that although 96% of dental students claimed to have the necessary ergonomic knowledge, only 58% were able to show correct working postures for different procedures, and only 29% were reported to be able to sit in the correct posture [4].

No study has yet been done at a South African dental school, to evaluate the levels of knowledge and practice regarding ergonomics among dental and dental hygiene students. There is anecdotal evidence from South African dental practitioners which suggests that many of them suffer from musculoskeletal pain as a result of incorrect ergonomic seating postures. As a result, it was decided to evaluate the ergonomic practices of undergraduate dental students to identify their level of knowledge and practice. This study aims to assess a baseline ergonomic knowledge, as well as highlight any shortcomings that may exist in the current dental school ergonomics training in South Africa.

## 2. Method

This was a cross sectional analytical study conducted at a tertiary dental institute in South Africa. All senior undergraduate dental (third, fourth and fifth year of study), and dental hygiene (DH) (second and third year of study) students registered in the 2021 academic year were invited to participate. The sample selection was restricted to students that performed clinical procedures only. Students in the earlier years of study were excluded, since their training is primarily restricted to theory. No sampling was done as all clinical dental students were invited to participate. In total, there were 202 students registered in 2021, and all were invited. The questionnaire was mailed four times to the students in order to improve the response rate.

The level of ergonomic knowledge was assessed through a modified version of a standardized questionnaire [4]. The questionnaire consisted of a set of 24 questions divided into three sections. The sections included were: a demographic section; a section on ergonomic knowledge; and a section on the prevalence and sites of musculoskeletal pain. The questionnaire was sent electronically to all invited students, together with a cover page indicating the rationale for the study and its objectives. The responses were anonymous and all data was confidential. The demographic section included the year of study and the degree that the students were registered for. The student's ergonomic knowledge consisted of three parts: their self-reported ergonomic knowledge; their theoretical knowledge; and their ability to practically implement this knowledge. The self-reported knowledge questions were of a dichotomous nature and included either a “yes,” “no” or “unsure” response. These questions were based on the self-assessment of ergonomic knowledge, their feelings about their ergonomic knowledge and its application, and their views as to how they were taught on this subject matter. Each question was scored as “1” for a positive response and a “0” for a negative/uncertain response. The sum of these scores resulted in the “total self-reported knowledge score” and was calculated out of a maximum score of 5 points. The scores were categorised into poor (less than 50%), moderate (51% to 69%), and good knowledge scores (70% or more).

The actual theoretical knowledge was assessed by indicating the ideal operator and patient positioning when treating different surfaces of various teeth. These questions were answered by marking the correct box with an “*X*”. Each correct answer was scored a “1”, and each incorrect or only partially correct answer was scored a “0”. This score consisted of three categories which included: the different operator positions; the patient's chair positioning; and the positioning of the patient's head in the dental chair. A score was calculated for each of these categories out of a maximum of 6 points, and the total theoretical knowledge score (which consisted of the sum of the 3 above) was assessed out of 18 points. The sum of these scores resulted in a final “total theory score” and were categorised into poor (less than 50%), moderate (50% to 69%), and good knowledge scores (70% or more).

The next section assessed the practical application of ergonomics amongst students. These questions were answered according to a three point Likert scale from always to never. The scores were calculated as follows: always positioned correctly-2 points; sometimes positioned correctly-1 point; never positioned correctly- 0 points. The sum of these scores were calculated to produce the “total practical score” out of a possible total of 20 points. The scores were categorised into poor (less than 50%), moderate (51% to 69%), and good knowledge scores (70% or more).

The third section related to the causes and prevalence of musculoskeletal pain. Through a series of multiple choice questions, participants were asked to indicate if, and where, they experienced pain. The final question was an open-ended question for comments and observations. The analysis of data was thematic in nature.

The data was collected using an electronic questionnaire and saved onto an excel spreadsheet. Data was then analysed using the statistical software package SPSS (Statistical Package for the Social Sciences), version 27. The quantitative data was analysed by means of mean, medians and frequencies, using basic statistical tests. Correlations were done by comparing mean scores against age, course of study, and year of study using ANOVA and Chi-square tests. The open-ended question was analysed using thematic approach and reported separately.

## 3. Results

There was a total of 106 respondents out of a sample of 202 registered dental students (response rate of 52%). The table below is a breakdown of the number of respondents per year of study and degree enrolled in 2021. (see Table1).

The ages ranged from 19 to 45 years with a mean age of 22.64 (SD ± 3.98) years. The majority of respondents (85%) were dental students. The mean self-reported ergonomic knowledge score was 3.39 out of 5 (68%; SD ± 1.15). Most of the DH students had either a fair or good score while half of the dental students (50%) had a score over 70% (Table 2).

The mean theoretical knowledge score based on different operator positions was 4.13 (69%; SD ± 1.01); the mean score based on the patients chair positioning was 4.76 (79%; SD ± 1.09), and the mean knowledge score regarding the positioning of the patient's head in the dental chair was 2.57 (43%; SD ± 1.00). Each of these was out of 6 points, and the total mean theoretical knowledge score (which consisted of the sum of the 3 above) was 11.40 (63%; SD ± 1.76). The majority (73%) of DH and 64% of dental students scored within the moderate category (Table 3).

The practical application of ergonomic knowledge was evaluated out of a maximum of 20 points. The mean score was 10.69 (53%; SD ± 1.70), and the majority of DH (93%) and dental (70%) students were classified within the moderate category. (see Table 4).

The most common problematic positions were leaning the neck forward (99%) and maintaining the head more than 30 cm from the patient's mouth (91%). In addition, 85% reported to place strain on the forearms, and 82% reported to not being able to keep the back straight.

There was no significant correlation between the theoretical knowledge and practical implementation of ergonomics. There was furthermore no significant correlation between theoretical knowledge and self-reported knowledge of ergonomics, nor was there a correlation between any of the categories and the year of study.

**Figure 1:**
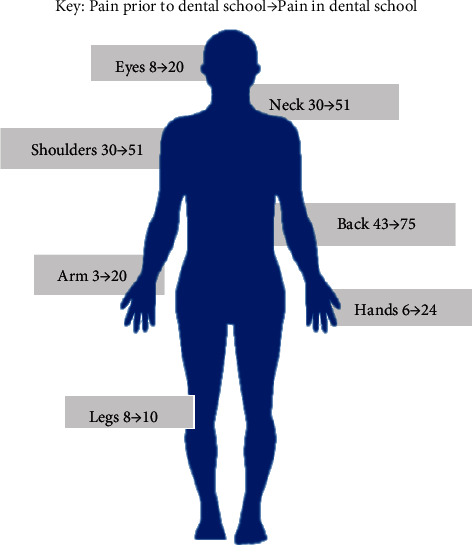
The prevalence of pain in various regions as experienced by dental students before and after participating in dental clinical activities.

Almost a third (32%) of respondents reported that they experienced pain prior to dental school, while 78% indicated that they were currently experiencing pain since they were in dental school.  Figure 1 shows the distribution of musculoskeletal pain and the change experienced in these sites before and after joining the dental school.

Most of the students (72%) perceived that musculoskeletal pain was related to poor posture, 57% felt that it was due to usage of electronic devices (cell phones, computers, tablets), and 11% considered previous trauma. Of all of the respondents, 40% reported to spend more than 7 hours per day while 42% spent between 4 and 6 hours per day utilizing electronic devices. However there was no significant correlation with the prevalence of pain and the duration of time spent on electronic devices.

No one responded to the open-ended section and hence the results were excluded.

## 4. Discussion

The response rate of this study was 52%, which was relatively low, and could have been due to the high workload as well as the time constraints of the students due to their upcoming examinations. Most of the respondents were dental students, and this could be due to the fact that the university trains more dental students per annum compared to DH students.

The mean self-reported knowledge score was 68%. This was rather poor considering that ergonomics is applicable in every clinical scenario. This score could imply that theoretical and practical training in ergonomics was under-emphasized amongst these students. While lectures on this topic have been given, it appeared that students neglected its implementation since they failed to realize its importance. Furthermore, it was possible that lecturers did not emphasize and physically demonstrate the correct seating posture to students sufficiently to the point where they felt competent in implementing it themselves.

The students' mean actual theoretical knowledge score was 63% which was similar to their self-reported knowledge score of 68%. Hence, it seems that students were aware that their ergonomic knowledge was only average, yet were not concerned about the lack of ergonomic knowledge. There is possibly insufficient lectures as well as theoretical and clinical assessments on ergonomics, hence the lack of concern as reported in another study [6]. Many studies have confirmed that the majority of under and postgraduate students leant their neck forward, did not maintain their head an appropriate distance from the patient's mouth, placed strain on the forearms and were unable able to keep their back straight [4, 7, 8].

One of the key dental disciplines in which incorrect posture is often maintained for prolonged periods of time, is represented by endodontics. This includes the extended use of both a magnifying glasses as well as a microscope, which may exacerbate the stress even further. The use of new generation instruments with increased stress resistance characteristics allows endodontic treatment to be performed in shorter times and hence should reduce muscle overload for the operators [9, 10].

All of these studies, including the current study reported, that while theoretical knowledge may have been acceptable, the practical implementation thereof, is an area of concern.

It was noted that the self-reported knowledge score was similar to the theoretical knowledge score. Hence, although the average student's ergonomic knowledge was only moderate, the students were aware of this. Since students did have some degree of knowledge of ergonomics, it was evident that some form of instruction had taken place, most likely through lectures. However, this knowledge was possibly under-emphasized, leading to these simple concepts being neglected and hence the relatively low average scores. It was furthermore evident, that there was a stark contrast in the student's theoretical knowledge and their ability to apply this knowledge. On average, students scored 63% in theory but only 53% were able to seat correctly. This could be that students found it difficult to apply theoretical knowledge practically, and that there was a possible lack of practical training and monitoring of posture by supervisors during clinical sessions.

The prevalence of musculoskeletal pain increased from 32% to 78% during their time at the dental school. This could be attributed to the physically taxing nature of the oral health profession and having to work in small spaces of the oral cavity for prolonged periods of time. This result was confirmed by another study which reported that 51% of dental students experienced pain already during their pre-clinical training [5]. In addition, the poor practical implementation of ergonomics by students as reported in this study, could have contributed to the increase in pain observed during clinical and pre-clinical sessions. The most common sites of pain in the current study were the back (75%), neck (51%), and shoulders (51%). This was expected considering that most of the strain tends to be placed upon these sites during dental procedures. These results concur with another study amongst dentists, which reported that more than 92% had some form of pain in the neck, lower back, and wrist [11]. This was significantly higher than what was currently observed, but could be due to the latter study being done on practicing dentists who had been working for a number of years compared to dental students. It is clear that musculoskeletal pain is a serious and devastating consequence, and has the potential to worsen with time if not addressed at the onset. The neck and back are areas that are consistently affected and can be due to the difficulty with visualization in dentistry and when manoeuvring to perform certain procedures.

The students reported that they felt that the most common causes of musculoskeletal pain were poor working posture (72%) and the use of electronics (57%). Almost half (40%) of the students reported to spend more than 7 hours per day on electronic devices, however this showed no significant correlation with the prevalence of pain as compared to students who spent less time on electronic devices. This could be due to the position in which students sit or relax and use their devices or could be confounded.

### 4.1. Limitations

This was a cross sectional study and hence the results cannot be seen as causative in nature. The students may have responded as anticipated and not according to their actual practice patterns (response bias). The junior students may have found it challenging to answer some of the questions due to their limited clinical experience, and confounding factors such as usage of electronics and previous injuries could have had a confounding effect on the results.

### 4.2. Recommendations

The number of lectures on ergonomics should be increased and consistently reinforced throughout the dental training program. Ergonomic practices should be assessed theoretically and practically as part of the clinical and pre-clinical procedures on a regular basis. Feedback should be given to students on how to improve and maintain correct ergonomic postures during each clinical session. Students should also be made aware of the risks associated with failure to adopt correct working postures, and be shown how to deal with muscular skeletal pain through stretching exercises.

## 5. Conclusion

Although students appeared to have a fair level of ergonomic knowledge, the practical implementation was lacking. The incidence of back, neck, and shoulder pain increased upon the commencement of dental school. It is vital that ergonomic training amongst undergraduate dental students be re-evaluated, and more emphasis be placed on it during the undergraduate training program.

## Figures and Tables

**Table 1:** Number of respondents per year of study and degree (*n* = 106).

Year of study	Degree	Number of respondents (*n* = 106)	Total number registered (*n* = 202) and (%) the response rate
2	Dental hygiene	11	11 (100)
3	Dental hygiene	6	9 (67)
	Dentistry	29	65 (45)
4	Dentistry	38	52 (73)
5	Dentistry	22	64 (34)
	Total	106	202 (52)

**Table 2:** The number of students to score between certain thresholds for the self-reported knowledge score (*n* = 102).

Category	DH N (%)	Dentistry N (%)	Total N (%)
Poor (<50%)	3 (20)	19 (22)	22 (22)
Moderate (51%–69%)	6 (40)	24 (28)	30 (29)
Good (>70%)	6 (40)	44 (50)	50 (49)
Total	15 (100)	87 (100)	102 (100)

**Table 3:** The number of students to score between certain thresholds for the theoretical knowledge score (*n* = 84).

Category	DH N (%)	Dentistry N (%)	Total N (%)
Poor (<50%)	0 (0)	5 (7)	5 (6)
Moderate (51%–69%)	8 (73)	47 (64)	55 (65)
Good (>70%)	3 (27)	21 (29)	24 (29)
Total	11 (100)	73 (100)	84 (100)

**Table 4:** The total practical application of ergonomics scores between certain thresholds (*n* = 100).

Categories	DH N (%)	Dentistry N (%)	Total N (%)
Poor (<50%)	1 (7)	20 (23)	21 (21)
Moderate (51%–69%)	13 (93)	60 (70)	73 (73)
Good (>70%)	0 (0)	6 (7)	6 (6)
Total	14 (100)	86 (100)	100 (100)

## Data Availability

The data is confidential and will be stored by the corresponding author in the Department of Community Dentistry.
